# Aspects épidémiologiques et anatomo-cliniques des tumeurs du cuir chevelu chez le noir africain

**DOI:** 10.11604/pamj.2019.34.98.18123

**Published:** 2019-10-17

**Authors:** Assane Diop, Moussa Diallo, Mame Tene Ndiaye, Biram Seck, Saer Diadie, Boubacar Ahy Diatta, Maodo Ndiaye, Zineb Baidouri, Ahmadou Deme, Fatimata Ly, Suzanne Oumou Niang, Assane Kane, Mame Thierno Dieng

**Affiliations:** 1 Dermatologie Hôpital Institut d'Hygiène Sociale de Dakar, Université Cheikh Anta Diop de Dakar, Dakar, Sénégal; 2 Dermatologie Hôpital Aristide Le Dantec, Université Cheikh Anta Diop de Dakar, Dakar, Sénégal; 3 Institut Curie Hôpital Aristide Le Dantec, Université Cheikh Anta Diop de Dakar, Dakar, Sénégal

**Keywords:** Tumeurs cuir chevelu, noir, carcinome épidermoïde, Scalp tumors, black, squamous cell carcinoma

## Abstract

**Introduction:**

Au Sénégal, les études portant sur les tumeurs du cuir chevelu sont quasi inexistantes. Notre objectif était de déterminer le profil épidémiologique et anatomo-clinique des tumeurs du cuir chevelu en dermatologie.

**Méthodes:**

Il s'agit d'une étude prospective, descriptive et analytique, réalisée sur une période de 16 mois (01 mars 2014 au 30 juin 2015), effectuée aux deux services de dermatologie de Dakar. La confirmation diagnostique était histopathologique.

**Résultats:**

Nous avions colligé 36 patients dont 14 cas de tumeurs malignes et 22 cas de tumeurs bénignes. Le sex ratio était de 1:1, et l'âge moyen de 51 ans pour les premières et 39 ans pour les dernières. La fréquence hospitalière était de 0,18%. Le délai moyen de consultation était de 14 mois pour les tumeurs malignes et 52 mois pour les formes bénignes. Dans 11 cas de tumeurs malignes, le traitement traditionnel était le premier recours. Les tumeurs malignes étaient: carcinome épidermoïde (n=8), carcinome basocellulaire (n=3), lymphome, hidradénocarcinome et dermatofibrosarcome de Darier Ferrand (un cas chacun). Les tumeurs bénignes étaient: botriomycome (n=5), kyste trichilemmal, hamartome sébacé, cylindrome et lipome (2 cas chacun), et chéloide, syringocystadénome papillifère, schwannome, neurofibrome et nævus (un cas chacun). La malignité de la tumeur était associée à l'aspect ulcéro-bourgeonnant (p=0.003), au diamètre >4cm (p=0.05), à la douleur (p=0.009) et au saignement (p=0.006).

**Conclusion:**

Les tumeurs du cuir chevelu, sur peau noire, sont dominées par les formes bénignes. Le carcinome épidermoïde est la tumeur maligne prédominante.

## Abstract (en)

**Introduction:**

In Senegal, the studies on scalp tumors are almost non-existent. The purpose of our study was to determine the epidemiological and anatomoclinical features of scalp tumors in dermatology.

**Methods:**

We conducted a prospective descriptive and analytical study in both Department of Dermatology at Dakar over a period of 16 months (01 March 2014-30 June 2015). Histopathological examination was used to confirm the diagnosis.

**Results:**

We collected data from the medical records of 36 patients, of whom 14 had malignant tumors and 22 benign tumors. Sex ratio was 1.1, the average age of patients with malignant tumors was 51 years while the average age of patients with benign tumors was 39 years. Hospitalization rate was 0.18%. The average time of consultation in patients with malignant tumors was 14 months, while in patients with benign tumors was 52 months. In 11 patients with malignant tumors, first treatment was based on traditional therapy. Malignant tumors included: squamous cell carcinoma (n=8), basal-cell carcinoma (n=3), lymphoma, hidradenocarcinoma and Darier-Ferrand dermatofibrosarcoma (one case for each type). Benign tumors included: botriomycoma (n=5), trichilemmal cyst, sebaceous hamartoma, cylindroma and lipoma (2 cases for each type), heloid, syringocystadenoma papilliferum, schwannoma, neurofibroma and nevus (one case for each type). Tumor malignancy was associated to ulcero-budding appearance (p=0.003), diameter >4 cm (p=0.05), pain (p=0.009) and bleeding (p= 0.006).

**Conclusion:**

Scalp tumors in black people are usually benign. Squamous cell carcinoma is the most common malignant form.

## Introduction

Les tumeurs du cuir chevelu sont des néoformations bénignes ou malignes, acquises ou congénitales. Elles se développent à partir des différents constituants de la peau du cuir chevelu [1]. Ces tumeurs posent un véritable problème diagnostique du fait de leur multiplicité. Seulement 2% des tumeurs épithéliales sont localisées au cuir chevelu [2]. Sur cette topographie, les tumeurs bénignes sont plus fréquentes [3]. Bien qu'elles sont dominées par les kystes trichilemmaux, leur prévalence exacte reste inconnue [3]. Sur phototype clair, les tumeurs malignes du cuir chevelu sont dominées par le carcinome basocellulaire [4,5]. Certaines de ces tumeurs malignes sont souvent agressives, avec une forte mortalité [6]. En Afrique subsaharienne les études sur les tumeurs du cuir chevelu sont très rares. L'objectif de notre travail était de préciser le profil épidémiologique et anatomo-clinique des tumeurs du cuir chevelu aux services de Dermatologie de Dakar.

## Méthodes

Il s'agissait d'une étude transversale, bicentrique, descriptive et analytique, avec un recueil prospectif des données. Cette étude était réalisée sur 16 mois (du 1er mars 2014 au 30 juin 2015), dans les deux services de référence en dermatologie de Dakar: Hôpital Aristide Le Dantec (HALD) et Hôpital Institut d'Hygiène Sociale (IHS). L'étude était réalisée dans le cadre d'un mémoire de fin d'études de spécialisation en dermatologie-vénérologie. Elle avait reçu l'approbation des responsables du diplôme d'études spécialisées en dermatologie-vénérologie du Sénégal. Les membres de cette équipe étaient constitués de 4 Professeurs, d'un Maître de Conférences agrégé et de 4 assistants. L'investigateur principal était une étudiante en fin d'études spécialisées en dermatologie-vénérologie. Elle était encadrée par un Assistant, sous la supervision d'un Professeur. Une fiche d'enquête était utilisée pour recueillir les variables. Ces dernières étaient socio-démographiques, cliniques et histopathologiques. Etait inclu tout patient consentant qui présentait une tumeur du cuir chevelu dont le diagnostic était évoqué par l'aspect clinique et confirmé à l'histopatholologie, après biopsie ou biopsie-exérèse. L'étude histopathologique était effectuée au laboratoire d'anatomie-pathologique de l'hôpital Aristide Le Dantec par un dermatopathologiste. Chaque patient pouvait se retirer de l'étude à tout moment sans que ça n'ait de conséquences sur la prise en charge de sa maladie. Les pathologies non dermatologiques étaient prises en charge en collaboration avec nos collègues des autres spécialités. Les données recueillies étaient saisies sur le logiciel Sphinx V5 et analysées grâce au logiciel Epi-info 7. Pour comparer les fréquences, le test de Khi 2 ou de Fischer étaient utilisés, avec un seuil de significativité si p<0,05.

## Résultats

Nous avions colligé 36 cas de tumeurs du cuir chevelu, comprenant 22 cas de tumeurs bénignes (61%) et 14 cas de tumeurs malignes (39%). La fréquence hospitalière était de 0,18%. Parmi les 96 tumeurs cutanées malignes observées dans ces deux services, durant cette période, la localisation au cuir chevelu représentait 14%. Pour les tumeurs malignes, la moyenne d"âge était de 51 ans avec des extrêmes de 8 et 76 ans, et concernant les tumeurs bénignes, la moyenne d'âge était de 39 ans avec des extrêmes de 15 et 72 ans. Les tumeurs bénignes étaient trouvées chez les patients âgés de moins de 50 ans dans 68,2% (n=15), et les tumeurs malignes l'étaient chez ceux âgés de plus de 50 ans dans 64,3%. Le sexe ratio des patients atteints de tumeurs bénignes était de 1,2 et ceux atteints de tumeurs malignes de 1. Une origine rurale était notée chez 78% des patients présentant une tumeur maligne et chez 63% des patients ayant une tumeur bénigne. Le Tableau 1 montre la répartition des tumeurs malignes du cuir chevelu selon les formes anatomo-cliniques. Le Tableau 2 montre les différents types de tumeurs bénignes du cuir chevelu chez nos patients.

**Tableau 1:** Répartition des 14 patients atteints de tumeurs malignes du cuir chevelu selon les formes anatomo-cliniques

Tumeurs malignes	Effectifs	Pourcentage (%)
Carcinome épidermoïde	8	57 %
Carcinome basocellulaire	3	21,4%
Lymphome	1	7%
Hidradénocarcinome	1	7%
Dermatofibrosarcome de Darier Ferrand	1	7%
Total	14	100,0

**Tableau 2:** Répartition des 22 patients atteints de tumeurs bénignes au cuir chevelu selon les formes anatomo-cliniques

Tumeurs bénignes	Effectifs	Pourcentage (%)
Kyste épidermoïde	6	27,2
Bourgeon charnu	5	22,7
Cylindrome	2	9,1
Lipome	2	9,1
Hamartome sébacé	2	9,1
Neurofibrome	1	4,5
Schwannome	1	4,5
Chéloïde	1	4,5
Syringocystadenome papillifère	1	4,5
Naevus	1	4,5
Total	22	100,0

**Figure 1:**
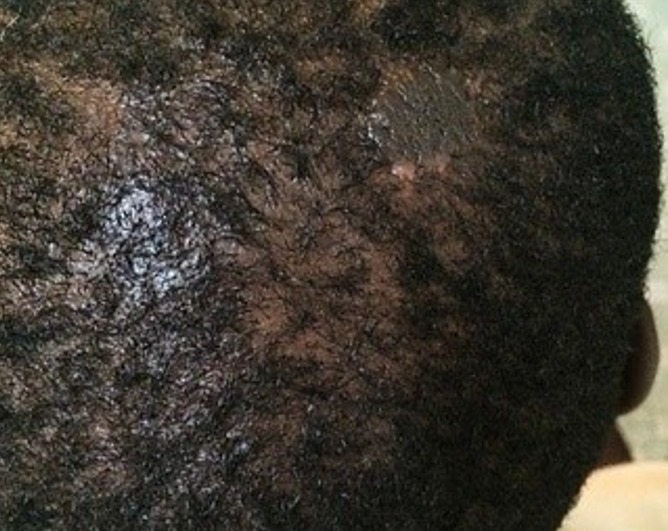
Naevus sébacé de Jadassohn du cuir chevelu

**Figure 2:**
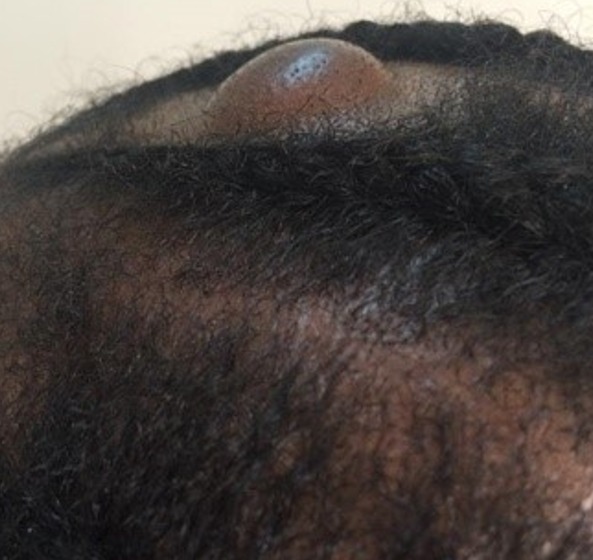
Kyste trichilemmal du cuir chevelu

**Figure 3:**
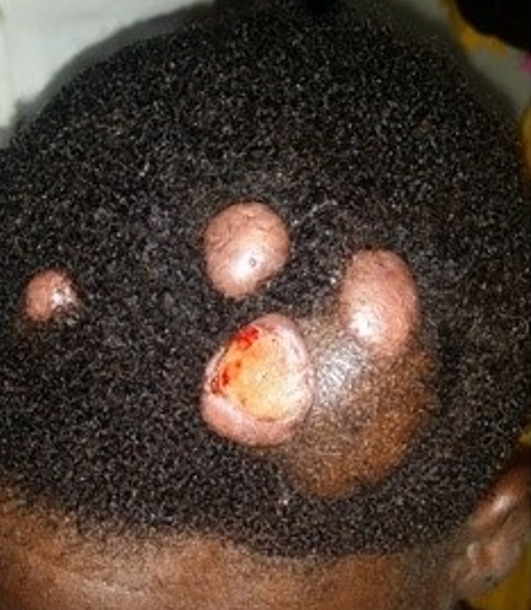
Cylindrome ulcéré du cuir chevelu

**Figure 4:**
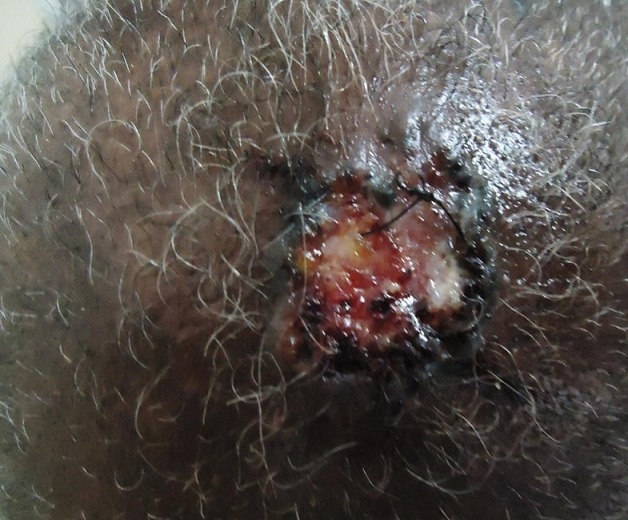
Carcinome basocellulaire du cuir chevelu

**Figure 5:**
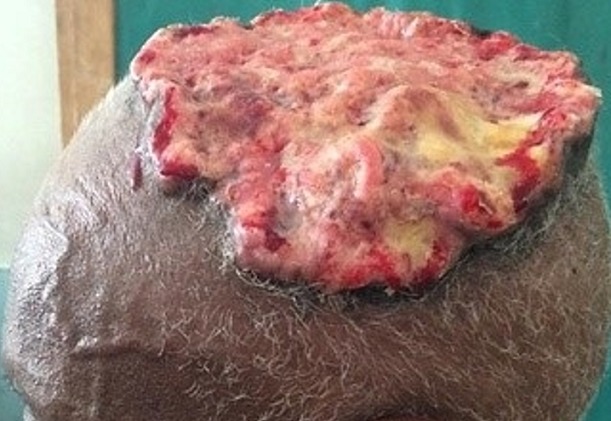
Hidradénocarcinome du cuir chevelu

**Figure 6:**
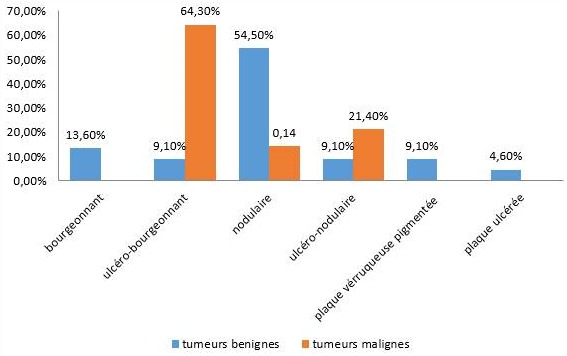
Répartition des tumeurs en fonction de l´aspect clinique des lésions

Le délai de consultation pour les tumeurs bénignes était compris entre 1 mois et 30 ans avec une moyenne de 52 mois. Pour les tumeurs malignes, ce délai était entre 2 mois et 4 ans avec une moyenne de 14 mois. Les figures Figure 1, Figure 2, Figure 3, Figure 4 et Figure 5 montrent quelques tumeurs du cuir chevelu retrouvées chez nos patients. Dans notre série, 42,8% (n=6) des tumeurs malignes étaient précédées de lésions précancéreuses. Ils s'agissaient de 5 cas de carcinome épidermoïde (CE) survenus sur des cicatrices de brûlures et post traumatiques dans 2 cas chacun, et une kératose actinique dans un cas. Le traitement traditionnel était le premier recours avant la consultation en dermatologie chez 11 patients présentant des tumeurs malignes, soit dans 78,6%. Les tumeurs malignes étaient ulcéro-bourgeonnantes dans 64,3% des cas et les tumeurs bénignes étaient nodulaires dans 54,5% (Figure 6). L'aspect ulcéro-bourgeonnant était associé à la malignité de la tumeur (p=0.033), et l'aspect nodulaire à la bénignité de la tumeur (p=0.008). La figure 6 montre les différents aspects cliniques des tumeurs bénignes et malignes chez nos patients. Un saignement était noté dans 22,7% des cas (n=5) de tumeurs bénignes et dans 71,4% (n=10) des cas de tumeurs malignes. La taille des tumeurs malignes variait entre 3 et 20 cm avec une moyenne de 10cm. Les tumeurs bénignes avaient une taille moyenne de 3cm avec des extrêmes de 1 et 6cm. Le Tableau 3 montre la différence statistique entre la malignité ou non de la tumeur et quelques variables cliniques. Des adénopathies cervicales d'allure tumorale étaient notées dans 64% (n=9) des tumeurs malignes. La radiographie du crâne et la TDM avaient objectivé des lésions ostéolytiques dans 4 cas de tumeurs malignes. Concernant les tumeurs malignes, une chirurgie était effectuée dans 50% (n=7) des cas et une chimiothérapie dans 42,9% (n=6). Il s'agissait de carboplastine associé au taxol dans deux cas, de 5 fluoracil associé à la cisplatine dans deux cas et de COP dans un cas. Pour les tumeurs bénignes, une biopsie exérèse était effectuée dans 59% (n=13) des cas, une exérèse dans 27% (n= 6), une abstinence dans 9% et une infiltration de corticoïdes dans 5%. Après un recul de 4 mois, l'évolution des tumeurs malignes était favorable dans deux cas, stationnaire dans un cas, et marquée par un décès dans 6 cas (trois cas de carcinomes épidermoïdes, deux cas de carcinomes basocellulaires et un cas de lymphome). Quatre cas étaient perdus de vue. Cette évolution était favorable dans 81% (n=18) des cas de tumeurs bénignes.

**Tableau 3:** Corrélation statistique entre le type de tumeur et les variables cliniques

Caractéristiques cliniques	Type de tumeur		P	OR [IC à 95%]
	Maligne	Bénigne		
**Délai de consultation**			0.322	
<12mois	9	10		
≥12mois	5	12		
Présence de douleur	8	3	0.009	7.85 [1.35-61.63]
Absence de douleur	6	19		
Saignement	10	5	0.006	7.9 [1.49-52.27]
Pas de saignement	4	17		
Prurit	5	5	0.396	
Pas de prurit	9	17		
**Aspects cliniques**			0.033	7.2 [1.29-40.05]
Nodulaire	2	12		
Autres	12	10		
Ulcéro-bourgeonnant	9	2	0.008	16 [2.37-199.3]
Autres	5	20		
**Taille**			0.05	15.6 [1.7-140]
< 4cm	1	12		
≥4cm	13	10		

## Discussion

Nous rapportons l'une des très rares études sur les tumeurs du cuir chevelu en dermatologie, en Afrique subsaharienne. Tous les 36 cas de tumeurs du cuir chevelu colligés, dans notre période d'étude (16 mois), étaient confirmés par un examen histopathologique. De surcroit, malgré l'effectif réduit, notre étude permet une analyse épidémioclinique comparative sur les tumeurs du cuir chevelu. Dans notre étude, la fréquence hospitalière en dermatologie des tumeurs du cuir chevelu était de 2,4 cas/mois. Récemment, une étude Indienne [3], effectuée dans un service d'anatomopathologie, avait trouvé 45 cas de tumeurs du cuir chevelu sur une période de 24 mois, soit une fréquence de 2 cas/mois. Par contre, une étude ghanéenne [7] avait retrouvé 10 cas sur une période de 6 ans dans un service de chirurgie plastique. La prédominance des tumeurs bénignes dans notre étude (61%) concorde avec les études indiennes de Leena *et al.* [3] et de Saikia *et al.* [8]. Dans la littérature, l'âge de survenue des tumeurs bénignes est plus précoce que celui des tumeurs malignes [3]. La tranche d'âge de 18 à 27 ans était la plus touchée par les tumeurs bénignes. Ce résultat est proche des données de l'étude de Leena *et al.* [3] qui avaient trouvé un âge compris entre 20 et 40 ans. L'âge jeune d'une de nos patients avec une tumeur maligne s'explique par sa génodermatose prénéoplasique (xeroderma pigmentosum) caractérisée par la survenue précoce de multiples cancers cutanés photo-induits [9]. Dans notre série, les tumeurs malignes du cuir chevelu représentaient 14% de toutes les tumeurs cutanées malignes. La topographie au cuir chevelu de ces tumeurs cutanées malignes est moins fréquente que celle de la face. En effet, dans l'étude de Diallo *et al.* [10], les tumeurs malignes étaient localisées à la face dans 19% des cas. Chez nos patients, le CE représentait plus de la moitié (57%) des cas de tumeurs malignes. Selon plusieurs auteurs, il est de loin le cancer cutané le plus fréquent sur peau noire qu'elle que soit la topographie [11-13]. Par contre, chez le sujet à peau claire, les cancers cutanés de l'extrémité céphalique sont dominés par le carcinome basocellulaire [13].

Dans la littérature, les facteurs de risque qui ont été incriminés dans l'apparition des tumeurs malignes du cuir chevelu sont: l'exposition solaire chronique, la présence de nævus sébacé, les antécédents de radiation ionisante, de brûlure, de traumatisme ou de chirurgie du cuir chevelu [1,6,14,15]. Dans la population africaine noire, les cancers de la peau ne sont pas souvent liés à l´exposition solaire, mais plutôt aux lésions cutanées précancéreuses (ulcères, cicatrices de brûlures) [11]. Parmi nos 8 cas de carcinomes épidermoïdes, 5 patients avaient des lésions précancéreuses (2 cas de cicatrices de brûlures, 2 cas de cicatrices post-traumatiques et un cas de kératose actinique). Nos résultats sont proches de ceux d'une petite série marocaine de 6 cas de CE au cuir chevelu où les lésions précancéreuses étaient constitués de 2 cas de cicatrices de brûlures et un cas de kératose actinique [15]. Pour autant, le fait que 80% de nos patients qui avaient une tumeur maligne soient d'origine rurale suggère l'existence d'un rôle de l'exposition solaire dans le développement des cancers du cuir chevelu. Par ailleurs, le dermatofibrosarcome de Darier Ferrand trouvé chez un adolescent de 16 ans est rarement rapporté. Dans l'étude de Kasse *et al.* [16], portant sur 22 cas de dermatofibrosarcome il n'existait aucune localisation au cuir chevelu. Katz *et al.* [5] avaient trouvé un seul cas de dermatofibrosarcome localisé au cuir chevelu sur un effectif de 197 cas. Dans l'étude de Chiu *et al.* [4], cette topographie représentait 2,8% (n=11) de leur série de 398 cas de dermatofibrosarcome. Pour les signes cliniques, le caractère ulcèro-bourgeonnant était plus fréquent en cas de tumeurs malignes, avec un lien statistiquement significatif (p= 0.0008). Ces résultats sont corroborés par une série marocaine portant sur 52 cas de tumeurs malignes du cuir chevelu [15]. De même, il existait une relation statistiquement significative (p=0,05) entre la taille de la tumeur et la malignité. Chez nos patients, cette grande taille des tumeurs malignes pourrait s'expliquer par le long délai de consultation (14 mois en moyenne). Ce retard diagnostique peut être dû à la négligence, l'inaccessibilité des structures sanitaires et le recours fréquent (78%) à la médecine traditionnelle. Par ailleurs, cette clinique assez caractéristique n'empêche que l'histopathologie reste incontournable dans la confirmation diagnostique. D'autant plus que nous avions trouvé un aspect ulcéro-bourgeonnant dans 9% des cas de tumeurs bénignes. Concernant ces tumeurs bénignes, le kyste épidermoïde était la plus fréquente (27,2%), suivi du bourgeon charnu (22,2%). En Inde, Leena *et al.* [3] avaient rapporté la même prédominance du kyste épidermoïde (28%) et des bourgeons charnus. Par contre, dans leur série les lipomes représentaient 5% et les nævus 2%. Cependant, le syringocystadénome papillifère, trouvé dans 4,5% des cas dans notre étude, constitue une tumeur annexielle rarement rapportée dans la littérature [17,18].

## Conclusion

Les tumeurs du cuir chevelu, sur peau noire, sont dominées par les formes bénignes. Le carcinome épidermoïde est la tumeur maligne prédominante. La mortalité élevée des formes malignes pourrait être expliquée par le retard diagnostique qui serait dû aux habitudes socioculturelles et à la sous-médicalisation.

### État des connaissances actuelles sur le sujet

- Les études sur les tumeurs du cuir chevelu sont très rares chez le noir africain.

### Contribution de notre étude à la connaissance

- Cher le noir africain, les tumeurs du cuir chevelu sont dominées par les formes bénignes;
- Le carcinome épidermoïde constitue la principale tumeur maligne du cuir chevelu. Dans la majorité des cas, il survient sur une dermatose préexistante;
- Le retard diagnostique explique la mortalité élevée des tumeurs maligne du cuir chevelu chez le noir africain.

## Conflits d’intérêts

Les auteurs ne déclarent aucun conflit d’intérêts.
